# Assessing the association of the *HNF1A *G319S variant with C-reactive protein in Aboriginal Canadians: a population-based epidemiological study

**DOI:** 10.1186/1475-2840-9-39

**Published:** 2010-08-18

**Authors:** Sylvia H Ley, Robert A Hegele, Philip W Connelly, Stewart B Harris, Mary Mamakeesick, Henian Cao, Joel Gittelsohn, Ravi Retnakaran, Bernard Zinman, Anthony J Hanley

**Affiliations:** 1 Department of Nutritional Sciences, University of Toronto, Toronto, Canada; 2 Robarts Research Institute and University of Western Ontario, London, Canada; 3 Department of Laboratory Medicine and Pathobiology, University of Toronto, Toronto, Canada; 4 The Keenan Research Centre of the Li Ka Shing Knowledge Institute, St Michael's Hospital, Toronto, Canada; 5 Center for Studies in Family Medicine, Schulich School of Medicine and Dentistry, University of Western Ontario, London, Canada; 6 Sandy Lake Health and Diabetes Project, Sandy Lake, Canada; 7 Center for Human Nutrition, Johns Hopkins Bloomberg School of Public Health, Baltimore, USA; 8 Division of Endocrinology, University of Toronto, Toronto, Canada; 9 Leadership Sinai Centre for Diabetes, Mount Sinai Hospital, Toronto, Canada; 10 Samuel Lunenfeld Research Institute, Mount Sinai Hospital, Toronto, Canada

## Abstract

**Background:**

C-reactive protein (CRP), a biomarker of inflammation, has been associated with increased risk of developing cardiovascular disease. Common variants of the hepatocyte nuclear factor 1A (*HNF1A) *gene encoding HNF-1α have been associated with plasma CRP in predominantly European Caucasian samples. *HNF1A *might therefore have an impact on vascular disease and diabetes risk that is mediated by CRP. In an Aboriginal Canadian population, a private polymorphism, *HNF1A *G319S, was associated with increased prevalence of type 2 diabetes. However, it has not been investigated whether this association is mediated by CRP. We aimed to investigate whether CRP was mediating the association between *HNF1A *G319S and type 2 diabetes in an Aboriginal Canadian population with a high prevalence of diabetes.

**Methods:**

A total of 718 individuals who participated in a diabetes prevalence and risk factor survey were included in the current analysis. Participants were genotyped for *HNF1A *G319S. Fasting plasma samples were analyzed for CRP. Fasting plasma glucose and a 75-g oral glucose tolerance test were obtained to determine type 2 diabetes.

**Results:**

The prevalence rate of type 2 diabetes was 17.4% (125/718) using the 1999 World Health Organization definition and was higher among S319 allele carriers compared to G/G homozygotes (p < 0.0001). Among participants without type 2 diabetes, CRP levels were higher among G/G homozygotes (1.64 [95% confidence interval 1.35-2.00] mg/l) than in S319 carriers (1.26 [1.04-1.54] mg/l) (p = 0.009) after adjustment for age, sex, 2-h post-load glucose, waist circumference, and serum amyloid A. CRP levels were elevated among those with diabetes after similar adjustment (4.39 [95% confidence interval 3.09-6.23] and 4.44 [3.13-6.30] mg/L, respectively), and no significant difference in CRP was observed between S319 carriers and non-carriers (p = 0.95).

**Conclusions:**

CRP levels were lower in S319 allele carriers of the *HNF1A *gene compared to non-carriers among individuals without diabetes, but this difference was not present among those with diabetes, who uniformly had elevated CRP levels. Therefore, while *HNF1A *appears to influence CRP concentrations in the non-diabetic state, chronic elevation of CRP is unlikely mediating the association between the *HNF1A *polymorphism and the high prevalence of type 2 diabetes in this Aboriginal population.

## Background

C-reactive protein (CRP), a biomarker of inflammation, has been associated with increased risk of developing cardiovascular disease [1,2]. In addition, plasma CRP concentrations have been associated with polymorphisms in the hepatocyte nuclear factor 1A (*HNF1A) *[3-5]. In a recent genome-wide association study, several loci including *HNF1A *which are involved in protein production related to insulin resistance, beta-cell function, weight gain, diabetes and/or early atherogenesis were associated with plasma CRP [4]. Based on these findings, the authors argued that variation in genes including *HNF1A *may have subsequent impact on vascular disease and diabetes influenced or marked by circulating CRP concentrations. In another genome-wide study, however, the authors reported that the *HNF1A *locus was not associated with coronary heart disease (CHD), although the locus was associated with CRP levels [5].

The *HNF1A *gene codes for a transcription factor, HNF-1α, which plays an important role in pancreatic beta-cell function [6-8]. In various populations, polymorphisms in the *HNF1A *gene are a common cause of maturity-onset diabetes of the young (MODY) [7-10], which is characterized by early age of onset and a marked defect in insulin secretion. CRP concentrations in MODY3 patients with severe loss-of-function mutations in *HNF1A *have not been systematically reported. In an Aboriginal Canadian population, a glycine to serine substitution at codon 319 (G319S) of the *HNF1A *gene was observed [11]. Unlike typical *HNF1A *variants in MODY3, the diabetes phenotype that emerged in this private polymorphism was non-MODY type 2 diabetes [11,12], although the S319 allele was associated with accelerated onset of type 2 diabetes in a gene-dosage manner in this population [12].

The recent genome-wide studies reported that variation in the *HNF1A *locus was associated with CRP concentrations [3-5], but not with CHD [5], suggesting that *HNF1A *is unlikely involved in CHD pathogenesis mediated by CRP. However, it has not been investigated whether plasma CRP mediates the association between the *HNF1A *polymorphism and type 2 diabetes. The aim of this study therefore was to determine whether CRP was mediating the association between *HNF1A *G319S and type 2 diabetes in an Aboriginal Canadian community known to have a high prevalence of type 2 diabetes [13].

## Methods

The Sandy Lake Health and Diabetes Project is a population-based study designed to determine diabetes rates and associated risk factors in an Aboriginal Canadian population. Between 1993 and 1995, data were obtained from 728 of 1018 (72%) eligible residents of Sandy Lake First Nation aged 10-79 years [13]. Signed and informed consent was obtained from all participants, and the study was approved by the Sandy Lake First Nation Band Council and University of Toronto Ethics Review Committee. After excluding participants with missing variables, 718 remained in the current analysis.

### Data collection and laboratory procedures

Blood samples were collected after an 8- to 12-h overnight fast to determine fasting glucose, insulin, lipid profile, CRP, and serum amyloid A (SAA) [13,14]. A 75-g oral glucose tolerance test was administered, and a second blood sample for glucose was drawn at 120 minutes post-load. Glucose concentration was determined using the glucose oxidase method. Insulin concentration was analyzed by radioimmunoassay (Pharmacia, Piscataway, NJ). Triglyceride, high-density lipoprotein (HDL) cholesterol and low-density lipoprotein (LDL) cholesterol were determined using standard methods described in the Lipid Research Clinics manual of operations [15]. CRP concentration was assessed using the Behring BN 100 and the N high-sensitivity CRP reagent (interassay CV 5.0% at 12.8 mg/l) (Dade-Behring, Mississauga, ON). SAA was determined using an enzyme-linked immunosorbent assay (interassay CV 11% at 81 mg/l) (BioSource International, Camarillo, CA). The homeostasis model assessment (HOMA) of insulin resistance (IR) and beta-cell function (beta) were estimated by the method of Matthews *et al. *[16]. Type 2 diabetes was defined by the revised 1999 World Health Organization (WHO) diagnostic criteria of fasting plasma glucose ≥7.0 mmol/l or 2-h postload plasma glucose ≥11.1 mmol/l.

Each anthropometric and blood pressure determination was performed twice, and the average was used in analyses. Waist circumference was measured at the iliac crest using an inelastic tape. Interviewers administered questionnaires to obtain demographic information.

### Genetic analysis

Restriction analysis with *Bse*DI followed by polyacrylamide gel electrophoresis was used to detect the DNA change underlying the *HNF1A *G319S amino acid variant [11]. DNA sequence-proven controls were run as standards for each genotyping reaction and a random 15% of samples were studied on another day with independent genotyping reactions. The concordance between replicates was 100%.

### Statistical analysis

Distributions of continuous variables were assessed for normality, and natural log transformations of skewed variables including CRP were used in statistical analyses. Descriptive statistics for continuous variables were summarized as mean ± standard deviation or median (25^th^-75^th ^percentile) for variables with a skewed distribution. Categorical variables were summarized using proportions. Characteristics of S319 allele carriers and non-carriers were compared using Welch's modified t test or chi-square test as appropriate. Fisher's exact test was used to assess *HNF1A *genotype frequencies. S/S319 and S/G319 genotypes were combined in subsequent analyses due to a small number of S/S319 carriers (n = 6) in the study population. For the purpose of analysis and discussion, individuals with S/S and S/G genotypes will be referred to as "S319 carriers".

To assess the association of the *HNF1A *G319S polymorphism with log CRP, least square means were calculated using general linear models: model 1, unadjusted; model 2, adjusted for age, sex, and log 2-h post-load glucose; model 3, adjusted for model 2 variables in addition to waist circumference; model 4, adjusted for model 3 variables and log SAA. Log SAA was added in the model 4 to assess whether the association between *HNF1A *and CRP was independent of another inflammatory marker.

The sex interaction with *HNF1A *G319S on log CRP levels was assessed by adding an interaction term to a model that included the G319S polymorphism with adjustment for age, sex, log 2-h post-load glucose, waist circumference, and log SAA. Data analyses were performed with the use of SAS software, version 9.2 (SAS Institute, Cary, NC), and with the consideration of two-sided p < 0.05 as statistically significant for all analyses.

## Results

Characteristics of study participants are presented in table 1according to the *HNF1A *G319S carrier status. There were no statistically significant differences between S319 allele carriers and non-carriers in metabolic risk variables, except for fasting and 2-hr post-load glucose levels and HOMA-beta (p < 0.05) (table 1).

**Table 1:** Characteristics of participants according to the *HNF1A* G319S carrier status

Characteristic	G/G319	S/G + S/S319	p
n (%)	554 (78.6)	151 (21.4)	
Age (years)*	30.0 ± 15.9	30.6 ± 15.4	0.70
Sex, male†	220 (39.7)	76 (50.3)	0.02
BMI (kg/m^2^)*	26.6 ± 5.90	27.0 ± 5.22	0.52
Waist circumference (cm)*	97.6 ± 14.7	99.1 ± 13.2	0.24
Hypertension, yes†‡	119 (21.5)	30 (19.9)	0.67
HDL cholesterol (mmol/l)*	1.25 ± 0.28	1.23 ± 0.27	0.46
LDL cholesterol (mmol/l)*	2.59 ± 0.74	2.56 ± 0.79	0.75
Triglyceride (mmol/l) §	1.25 (0.88-1.71)	1.27 (0.97-1.87)	0.06
Fasting glucose (mmol/l) §	5.4 (5.1-5.9)	5.6 (5.0-7.6)	< 0.0001
2-hour postload glucose (mmol/l) §	5.7 (4.4-7.2)	6.0 (4.8-9.8)	0.002
HOMA-insulin resistance§	3.61 (2.38-6.27)	3.96 (2.16-6.87)	0.44
HOMA-beta§	144.4 (98.3-204.5)	110.3 (67.8-180.5)	0.0004
C-reactive protein (mg/l) §	2.17 (0.63-5.73)	1.85 (0.57-5.05)	0.29
Serum amyloid A (mg/l) §	7.71 (4.73-12.98)	7.18 (5.03-11.39)	0.48

Abbreviation: BMI, body mass index; HDL high-density lipoprotein; LDL low-density lipoprotein; HOMA, homoeostasis model assessment. N of subjects for each characteristic varying slightly due to occasional missing values. *Mean ± standard deviation and Welch's t test performed. †n (%) and Chi-Square test performed. ‡Hypertension is defined as a systolic blood pressure ≥130 mmHg or diastolic blood pressure of ≥85 mmHg or receiving antihypertensive medication therapy. §Medians (25^th^-75^th ^percentile) and Welch's t test performed on log transformation.

The prevalence rate of type 2 diabetes was 17.4% (125/718) using the 1999 WHO definition and was higher among S319 allele carriers compared to non-carriers (p < 0.0001) (table 2). Individuals with diabetes were older and more likely to have hypertension and they had lower HDL cholesterol and HOMA-beta and higher adiposity measures, LDL cholesterol, triglyceride, fasting and 2-h postload glucose, HOMA-IR, CRP, and SAA (all < 0.001; see additional file 1: table S1-characteristics of participants according to the diabetes status).

**Table 2:** *HNF1A *genotype frequencies and prevalence of type 2 diabetes using 1999 WHO definition

	G/G319	S/G319	S/S319	p
Total, n (%)	554 (78.6)	145 (20.6)	6 (0.9)	< 0.0001
Diabetes, n (%)	77 (13.9)	42 (29.0)	5 (83.3)	<0.0001

Fisher's exact test was performed.

In individuals without type 2 diabetes, CRP levels of S319 carriers and non-carriers were compared (table 3). In the unadjusted model, CRP was higher among S319 non-carriers compared to carriers (p = 0.02). After adjusting for age, sex, log 2-h post-load glucose, waist circumference, and log SAA, least square mean CRP levels remained higher among S319 non-carriers (1.64 [95% confidence interval 1.35-2.00] mg/l) than in S319 carriers (1.26 [1.04-1.54] mg/l) (p = 0.009) (table 3). Additionally adjusting the model 4 for HDL cholesterol, LDL cholesterol, hypertension, triglyceride, smoking history, HOMA-IR, or HOMA-beta did not change this association (beta effect size change ≤3% for each model) (data not shown).

**Table 3:** Concentrations of CRP according to the *HNF1A *G319S carrier status among participants without diabetes

	Beta	SE	CRP [95% CI] (mg/L) *		p
				
			G/G319	S/G + S/S319	
Model 1†	-0.3525	0.1498	1.65 (1.23-2.22)	1.16 (0.93-1.46)	0.02
Model 2‡	-0.2803	0.1351	1.65 (1.26-2.15)	1.24 (0.95-1.62)	0.04
Model 3§	-0.3310	0.1215	1.66 (1.31-2.11)	1.19 (0.94-1.51)	0.007
Model 4∥	-0.2635	0.1008	1.64 (1.35-2.00)	1.26 (1.04-1.54)	0.009

*Least square mean CRP values are back-transformed after using log-transformed CRP to calculate p-values. Group sample sizes of 477 (G/G319) and 104 (S/G + S/S319) have 90.8% power to detect a difference between mean CRP concentrations ± standard deviations of 1.65 (G/G319) ± 1.39 and 1.16 (S/G + S/S319) ± 1.36 at a significance level of 0.05. †Model 1: unadjusted ‡Model 2: adjusted for age, sex, log 2-h post-load glucose §Model 3: adjusted for model 2 variable and waist circumference ∥Model 4: adjusted for model 3 variables and log serum amyloid A

Among individuals with type 2 diabetes, CRP levels were not significantly different between S319 non-carriers and carriers when adjusted for age, sex, log 2-h post-load glucose, waist circumference, and log SAA (4.44 [95% CI 3.13-6.30] and 4.39 [3.09-6.23] mg/L, respectively; p = 0.95) (all other models p > 0.05; table 4).

**Table 4:** Concentrations of CRP according to the *HNF1A *carrier status among participants with diabetes

	Beta	SE	CRP [95% CI] (mg/L)*		p
				
			G/G319	S/G + S/S319	
Model 1†	-0.2162	0.1847	4.79 (3.33-6.88)	3.86 (2.69-5.54)	0.24
Model 2‡	-0.3514	0.2354	5.05 (3.18-8.01)	3.52 (2.22-5.58)	0.13
Model 3§	-0.2971	0.2286	4.96 (3.17-7.77)	3.69 (2.36-5.77)	0.20
Model 4∥	-0.0109	0.1786	4.44 (3.13-6.30)	4.39 (3.09-6.23)	0.95

*Least square mean CRP values are back-transformed after using log-transformed CRP to calculate p-values. Group sample sizes of 77 (G/G319) and 47 (S/G + S/S319) have 99.9% power to detect a difference between mean CRP concentrations ± standard deviations of 4.79 ± 1.03 (G/G319) and 3.86 ± 0.93 (S/G + S/S319) at a significance level of 0.05. †Model 1: unadjusted ‡Model 2: adjusted for age, sex, log 2-h post-load glucose §Model 3: adjusted for model 2 variable and waist circumference ∥Model 4: adjusted for model 3 variables and log serum amyloid A

There was no sex interaction with the *HNF1A *G319S polymorphism on CRP levels (p > 0.05).

## Discussion

In diabetes-free individuals, plasma CRP levels were lower among the S319 allele carriers compared to non-carriers, although both group mean CRP levels were within the average risk range for cardiovascular events (1.0-3.0 mg/L) [17]. Among individuals with type 2 diabetes, however, CRP levels were elevated to the high risk range for cardiovascular events (> 3 mg/L) [17]in both S319 allele carriers and non-carriers. In addition, the difference in CRP levels between the S319 carrier statuses was not present in those with diabetes, who are likely under chronic inflammatory stress.

Genetic variations in the *HNF1A *gene encoding HNF-1α protein have been associated with plasma CRP concentrations in predominantly European Caucasian samples [3-5]. In a genome-wide association study, several loci including *HNF1A *which are involved in protein production related to insulin resistance, beta-cell function, weight gain, diabetes and/or early atherogenesis were associated with plasma CRP [4]. Therefore, the authors argued that variation in genes including *HNF1A *may have subsequent impact on vascular disease and diabetes risk that is influenced or marked by circulating CRP concentrations. In another genome-wide study, however, it was reported that the *HNF1A *locus was not associated with CHD although the locus was associated with CRP levels [5], arguing against a causal association of CRP with CHD.

Although the association of CRP with increased risk of developing cardiovascular disease has been well documented [1,2,5], previous studies on the association of CRP with incident type 2 diabetes have been less consistent. Several studies associated increased baseline levels of CRP [18,19]with incident type 2 diabetes, while others reported no association, including a report from the current study population [20-22]. We have previously reported a higher prevalence of type 2 diabetes among *HNF1A *S319 carriers compared to non-carriers in this population [11], which is paradoxical in the context of the current finding of S319 carriers having a low level of CRP. The higher prevalence of type 2 diabetes observed among S319 carriers is likely explained by the compromised ability of these subjects to mount an adequate insulin response, resulting in an earlier loss of glycemic control [12].

HNF-1α protein binding to promoter regions of the CRP gene, down stream of the IL-6 responsive site, is known to be involved in synergistic trans-activation of CRP promoter [23-25]. The *HNF1A *G319S genomic sequence was recently reported to give rise to two abnormal transcripts, with altered quantities of the normal splicing products and reduced total *HNF1A *transcript levels [26]. Therefore, under the acute-phase signaling mechanism, abnormal HNF-1α produced by S319 carriers may have an impact on CRP production explaining the lower CRP level observed among non-diabetic individuals with the *HNF1A *G319S polymorphism. Among individuals with diabetes who are likely under chronic inflammatory stress, however, another mechanism may be modulating increased circulating CRP. Stimulation of the CRP promoter caused by increased circulating IL-6 and/or other signaling factors involved may be responsible for the increase in CRP levels in diabetic subjects [27], while the synergistic effects of HNF-1α contributing to CRP expression would be masked in this setting [23-25].

Since ethnic variation in polymorphisms within the coding regions of HNFs has been reported in non-European populations [28-30], it was important to confirm the association of the population-specific polymorphism, *HNF1A *G319S, with CRP. The current study provides evidence that variation in *HNF1A *is associated with variation in CRP in diabetes-free individuals (although not in those with diabetes) in populations other than European Caucasians.

## Limitations

Our study was conducted within an isolated Aboriginal community. Although this allowed us to investigate a population-specific polymorphism, this also limited our sample size. Therefore, it is recommended that our study results be confirmed in future studies with larger sample sizes, especially the observation in individuals with diabetes. Furthermore, it would have been beneficial to assess other biomarkers (e.g. HNF-1α protein) in addition to CRP. Because our data collection was completed in 1993-1995 followed by biochemical assays, our current data analysis was limited to previously assayed biomarkers.

## Conclusions

In summary, CRP levels were lower in *HNF1A *S319 allele carriers compared to non-carriers among Aboriginal Canadians without diabetes, but this difference was not present among those with prevalent type 2 diabetes, a group with markedly elevated CRP levels. Therefore, while *HNF1A *appears to influence CRP concentrations in the non-diabetic state, chronic elevation of CRP is unlikely mediating the association between the *HNF1A *polymorphism and the high prevalence of 2 diabetes in this Aboriginal population.

## Competing interests

The authors declare that they have no competing interests

## Authors' contributions

All of authors read and approved the final manuscript and contributed in revising the manuscript critically for important intellectual content. SHL contributed to the statistical analysis, interpretation of the data and drafted the manuscript. RAH and HC contributed to the genetic analysis and interpretation of the data. MM contributed to the acquisition of data. RAH, PWH, SBH, JG, RR, BZ and AJH contributed to the conception and design of the study.

## Supplementary Material

Additional file 1 Table S1: Characteristics of participants according to the diabetes status Click here for file
