# Supplementary material for: Assessing the association of the HNF1A G319S variant with C-reactive protein in Aboriginal Canadians: a population-based epidemiological study
Source: Cardiovasc Diabetol. 2010 Aug 18;9:39. doi: 10.1186/1475-2840-9-39 (PMC2929219; doi:10.1186/1475-2840-9-39)
Supplement: Additional file 1 — Table S1: Characteristics of participants according to the diabetes status [file 1475-2840-9-39-S1.DOC]

**Additional file**

Additional Table 1. Characteristics of participants according to the diabetes status

| Characteristic | No Diabetes | Diabetes | p |
| --- | --- | --- | --- |
| n (%) | 593 (82.6) | 125 (17.4) |  |
| Age (years)* | 27.2 ± 14.6 | 43.4 ± 14.7 | <0.0001 |
| Sex, male† | 253 (42.7) | 49 (39.2) | 0.48 |
| BMI (kg/m^2^)* | 25.9 ± 5.67 | 30.3 ± 4.64 | <0.0001 |
| Waist circumference (cm)* | 96.0 ± 14.3 | 106.7 ± 11.0 | <0.0001 |
| Hypertension, yes†‡ | 107 (17.7) | 45 (36.0) | <0.0001 |
| HDL cholesterol (mmol/l)* | 1.26 ± 0.28 | 1.17 ± 0.28 | 0.0005 |
| LDL cholesterol (mmol/l)* | 2.49 ± 0.73 | 2.98 ± 0.72 | <0.0001 |
| Triglyceride (mmol/l) § | 1.16 (0.85-1.57) | 1.89 (1.46-2.40) | <0.0001 |
| Fasting glucose (mmol/l) § | 5.3 (5.0-5.7) | 9.5 (7.4-15.5) | <0.0001 |
| 2-hour postload glucose (mmol/l) § | 5.4 (4.3-6.6) | 14.5 (11.5-18.5) | <0.0001 |
| HOMA-insulin resistance§ | 3.19 (2.13-4.72) | 9.15 (6.69-13.80) | <0.0001 |
| HOMA-beta§ | 148.1 (105.0-206.0) | 70.4 (29.4-120.2) | <0.0001 |
| C-reactive protein (mg/l) § | 1.65 (0.48-4.87) | 5.00 (2.33-7.84) | <0.0001 |
| Serum amyloid A (mg/l) § | 7.07 (4.46-11.59) | 9.95 (6.69-15.71) | <0.0001 |

Abbreviation: BMI, body mass index; HDL high-density lipoprotein; LDL low-density lipoprotein; HOMA, homoeostasis model assessment. N of subjects for each characteristic varying slightly due to occasional missing values. *Mean ± standard deviation and Welch’s t test performed. †n (%) and Chi-Square test performed. ‡Hypertension is defined as a systolic blood pressure ≥130 mmHg or diastolic blood pressure of ≥85 mmHg or receiving antihypertensive medication therapy. §Medians (25^th^-75^th^ percentile) and Welch’s t test performed on log transformation.
